# A predictive model based on the GLIM diagnosis for malnutrition in older adult heart failure patients

**DOI:** 10.3389/fnut.2025.1551483

**Published:** 2025-05-30

**Authors:** Xinyu Tang, Changying Zhang, Feng Xiao, Fang Yang, Xiaoya Zhu, Yunlai Gao

**Affiliations:** Department of Cardiology, Wuxi People's Hospital Affiliated to Nanjing Medical University, Wuxi, China

**Keywords:** heart failure, GLIM, malnutrition, predictive model, nomogram

## Abstract

**Background and aims:**

Malnutrition is closely associated with adverse clinical outcomes in older adult heart failure (HF) patients. Currently, there is a distinct absence of specific diagnostic tools to identify malnutrition within this particular population. Therefore, this study aims to analyze the factors influencing malnutrition in older adult HF patients based on the Global Leadership Initiative on Malnutrition (GLIM) criteria, with the goal of developing a rapid and accurate diagnostic method to identify malnutrition.

**Methods:**

The research incorporated a primary cohort study of 163 HF patients aged 65 and above and a validation cohort of 69 patients. The nutritional status of these patients was assessed according to the GLIM criteria. Logistic regression analysis was conducted to determine the independent risk factors of malnutrition. Subsequently, a nomogram model was developed and validated.

**Results:**

According to the GLIM criteria, 54 patients (33.1%) and 22 patients (32.4%) in two patient cohorts were suffering from malnutrition. The logistic analyses revealed that body mass index (BMI), grip strength, mid-upper arm circumference (MUAC), fat-free mass (FFM), and albumin independently serve as risk factors of malnutrition in older adult HF patients. The nomogram model demonstrates excellent discriminative ability, with an area under the curve (AUC) of 0.921 (95% CI: 0.881–0.962). While the AUC of validation cohort is 0.899 (95% CI: 0.827–0.972).

**Conclusion:**

In older adult HF patients, BMI, grip strength, FFM, MUAC and albumin are identified as independent risk factors for malnutrition. The constructed nomogram based on these factors can accurately predict malnutrition and holds significant practical value.

## 1 Introduction

Malnutrition is a prevalent issue among older adult HF patients, correlating with increased rates of complications and mortality, as well as reduced treatment efficacy (1–3). Timely nutritional interventions can enhance the clinical outcomes of HF patients (4). Hence, both the European Society of Cardiology (ESC) Heart Failure Guidelines and the AHA/ACC/HFSA Guideline for the Management of Heart Failure advocate for the monitoring and prevention of malnutrition in HF patients (5, 6). However, the lack of specific tools for diagnosing malnutrition in older adult HF patients leads to underdiagnosis of malnutrition in this population, making timely intervention challenging (7). Therefore, there is an urgent need to develop diagnostic tools specific to malnutrition in older adult HF patients, to facilitate precise diagnosis of malnutrition and prompt implementation of nutritional interventions.

In 2018, the GLIM criteria were established for diagnosing malnutrition (8). The effectiveness of its diagnosis of malnutrition has been proven in many diseases, including older adult community residents (9), cancer (10) and cardiovascular diseases (11). Meanwhile, a recent study has shown that the use of GLIM criteria to diagnose malnutrition in older adult HF patients also has good diagnostic performance (12).

In clinical practice, the utilization of the GLIM criteria for diagnosis involves numerous parameters, rendering the process relatively complex and time-consuming (13). Upon admission, utilizing a simplified method for rapid malnutrition diagnosis (while still achieving high accuracy) can facilitate early nutritional intervention for older adult heart failure patients, thus improving prognosis. Therefore, this study aims to develop and validate a simple, rapid, and accurate diagnostic method for identifying malnutrition in older adult HF patients.

## 2 Methods

### 2.1 Study design and participants

This is an observational research. The primary cohort comprised 163 heart failure patients aged 65 years and older, admitted to Wuxi People’s Hospital between May 2022 and May 2023. Exclusion criteria included: (1) history of cardiac transplant, (2) left ventricular pacemaker treatment, or assist device; (3) chronic hemodialysis or peritoneal dialysis; (4) acute myocarditis. Furthermore, from June 2023 to January 2024, an independent cohort of older adult heart failure patients meeting the same inclusion and exclusion criteria was recruited at Wuxi People’s Hospital to form the validation cohort for this study. All assessments and data collection were conducted post-admission. Participants were notified of their participation in the study and of their right to withdraw at any time. The sample size calculation was conducted using PASS software. According to previous literature (12), the incidence of malnutrition in older adult heart failure patients is 42.4%. A type I error was set at 0.05 and a type II error was set at 0.2, resulting in a calculated sample size of 102. Ultimately, a total of 163 patients were included in the primary cohort of this study.

### 2.2 The steps to define malnutrition

A two-step approach is employed to define malnutrition following the GLIM criteria (8). Initially, the Malnutrition Universal Screening Tool (MUST) was employed (14), as recommended by the GLIM consensus paper, to screen for malnutrition risk. Patients with a MUST score of 1 or higher were identified as at risk of malnutrition. Subsequently, a comprehensive assessment of patients at risk of malnutrition was conducted following the GLIM standards. The diagnostic criteria for malnutrition consist of three phenotypic and two etiological components, with each component required to meet at least one criterion to confirm malnutrition. The weight loss phenotypic criteria was determined as a weight loss of >5% within the past 6 months or >10% over a period longer than 6 months in HF patients. The decrease in muscle mass was determined by the appendicular skeletal muscle index, assessed using bioelectrical impedance analysis (BIA), utilizing the cut-off values recommended by the Asian Working Group on Sarcopenia (7 kg/m^2^ for males and 5.7 kg/m^2^ for females) (15). One of the phenotypic criteria includes age <70 years with BMI < 18.5 kg/m^2^ or age >70 years with BMI < 20 kg/m^2^. The etiological components of the GLIM criteria include (a) a reduced intake of less than 50% of energy requirements for a duration exceeding 1 week or any reduction lasting over 2 weeks, and (b) the presence of chronic inflammatory pathology. Malnutrition is diagnosed when at least one phenotypic criterion and one etiological criterion are present.

### 2.3 Measurement of MUAC

Measurement of MUAC involves the patient’s left arm being in a naturally hanging position. The circumference is then measured twice at the midpoint of the left upper arm, and the average of these measurements is taken as the patient’s MUAC (16).

### 2.4 Development of the nomogram

We take the bidirectional stepwise selection method by utilizing the Akaike information criterion to perform a multivariable logistic regression analysis on the cohort of HF patients to find independent risk factors for malnutrition. Variables with a significance level of *p* < 0.05 were identified as independent predictors. The forest plot was generated to depict the significance and accuracy of the predictive variables, which were then integrated into the development of an individualized malnutrition prediction nomogram. In the Cartesian coordinate system, we centered on an invalid vertical line (with an x-coordinate scale of 1) and described each effect size and confidence interval included in the study with multiple line segments parallel to the x-axis, ultimately creating a forest plot. For validating the nomogram, the study computed the AUC using 1,000 bootstrap iterations to measure discrimination and executed 1,000 bootstrap iterations for calibration curve analysis to assess the calibration. The discriminatory ability of the model is assessed using the C-index. Typically, a C-index ranging from 0.51 to 0.70 indicates moderate accuracy, while a value between 0.71 and 0.90 demonstrates moderate to high accuracy, and values greater than 0.90 are interpreted as indicating high accuracy. In predictive modeling, the Area Under the Curve (AUC) is equivalent to the C-index. The Hosmer and Lemeshow tests were used to statistically assess Calibration. The clinical efficacy of nomogram was assessed using decision curve analysis (DCA) to calculate the standardized net benefit at various probability thresholds. The GLIM standard was used as a benchmark to evaluate the nomogram model.

### 2.5 Statistics analysis

Continuous variables were reported as mean ± standard deviation or as median (interquartile range, IQR), depending on the normality of the data distribution. Differences in group means or medians were examined through *t*-tests or the Mann–Whitney *U* test. In the analysis of categorical variables, frequencies and percentages were presented, with group comparisons carried out using the Pearson chi-square test. The outcomes were presented as odds ratios (ORs) along with 95% confidence intervals (CIs). A Spearman correlation analysis (rs) was performed to evaluate the relationship between two quantitative variables (17). The statistical significance level for all analyses was established at *p* < 0.05 in a two-tailed test. Statistical analysis was conducted using IBM SPSS Statistics version 25.0 (New York, USA).

### 2.6 Ethical statement

The study obtained approval from the Ethics Review Committee of Wuxi People’s Hospital (Ethical Approval Number: 2023KY23114). All participants provided written informed consent prior to the initiation of the study.

## 3 Results

### 3.1 Baseline characteristics

As shown in Table 1, the primary cohort included a total of 163 older adult inHF patients (58.9% male, 41.1% female). Among them, 54 patients (33.1%) met the criteria for malnutrition as defined by the GLIM, while 109 patients (66.9%) had normal nutritional status. The two groups have no statistically significant differences, regarding age, gender, heart rate, blood pressure, heart function classification, comorbidities, and NT-proBNP (*p* > 0.05). However, significant differences were observed in ASMI, weight, BMI, grip strength, fat-free mass (FFM), mid-upper arm circumference (MUAC), and albumin levels (*p* < 0.05). The baseline characteristics of the validation cohort closely resembled those of the primary cohort.

**Table 1:** Patient characteristics stratified by malnutrition as defined by the GLIM criteria.

Variables	GLIM diagnosis					
	Primary cohort by GLIM diagnosis *n* = 163			Validation cohort by GLIM diagnosis *n* = 68		
	Without malnutrition (*n* = 109)	With malnutrition (*n* = 54)	*p* value	Without malnutrition (*n* = 46)	With malnutrition (*n* = 22)	*p* value
Sex, male, *n* (%)	64 (58.7%)	32 (59.3%)	0.947	30 (65.2%)	12 (54.5%)	0.397
Age, mean ± sd	76.9 ± 6.9	76.5 ± 6.8	0.763	72.1 ± 6.0	73.1 ± 7.0	0.518
BMI, median (IQR)	23.8 (21.3, 26.7)	19.1 (17.8, 20.8)	*p* < 0.001	25.3 (23.1, 28.3)	19.0 (18.1, 22.3)	*p* < 0.001
ASMI (kg/m^2), median (IQR)	7.5 (6.3, 8.3)	6.7 (5.6, 7.4)	*p* < 0.001	8.0 (7.0, 8.3)	6.7 (5.3, 7.0)	*p* < 0.001
NYHA Class, *n* (%)			0.274			0.354
IV	56 (34.4%)	26 (16%)		18 (26.5%)	12 (17.6%)	
III	44 (27%)	19 (11.7%)		23 (33.8%)	10 (14.7%)	
II	9 (5.5%)	8 (4.9%)		4 (5.9%)	0 (0%)	
I	0 (0%)	1 (0.6%)		1 (1.5%)	0 (0%)	
Systolic blood pressure (mmHg), mean ± sd	133.0 ± 21.9	128.52 ± 22.9	0.223	132.7 ± 23.4	125.0 ± 22.0	0.201
Diastolic blood pressure (mmHg), mean ± sd	76.4 ± 13.2	75.0 ± 11.8	0.509	80.8 ± 15.5	74.4 ± 13.5	0.101
Heart rate (bpm), mean ± sd	82.3 ± 18.8	86.2 ± 19.6	0.221	88.7 ± 17.4	87.0 ± 19.3	0.718
LVEF (%), mean ± sd	51.6 ± 11.4	46.1 ± 12.9	0.006	44.6 ± 10.1	45.5 ± 10.8	0.759
Heart failure phenotypes, *n* (%)			0.030			0.866
HFpEF	63 (38.7%)	20 (12.3%)		17 (25%)	7 (10.3%)	
HFmrEF	22 (13.5%)	13 (8%)		17 (25%)	8 (11.8%)	
HFrEF	24 (14.7%)	21 (12.9%)		12 (17.6%)	7 (10.3%)	
Comorbidities (%)						
Atrial fibrillation, *n* (%)			0.165			0.62
No	44 (27%)	28 (17.2%)		28 (41.2%)	12 (17.6%)	
Yes	65 (39.9%)	26 (16%)		18 (26.5%)	10 (14.7%)	
Hypertension, *n* (%)			0.612			0.888
No	36 (22.1%)	20 (12.3%)		18 (26.5%)	9 (13.2%)	
Yes	73 (44.8%)	34 (20.9%)		28 (41.2%)	13 (19.1%)	
Coronary artery disease, *n* (%)			0.953			0.492
No	51 (31.3%)	25 (15.3%)		25 (36.8%)	10 (14.7%)	
Yes	58 (35.6%)	29 (17.8%)		21 (30.9%)	12 (17.6%)	
Diabetes, *n* (%)			0.478			0.754
No	77 (47.2%)	41 (25.2%)		29 (42.6%)	13 (19.1%)	
Yes	32 (19.6%)	13 (8%)		17 (25%)	9 (13.2%)	
COPD, *n* (%)			0.467			0.821
No	97 (59.5%)	50 (30.7%)		44 (64.7%)	20 (29.4%)	
Yes	12 (7.4%)	4 (2.5%)		2 (2.9%)	2 (2.9%)	
Prescription at discharge						
Loop diuretics, *n* (%)			0.637			0.795
No	17 (10.4%)	10 (6.1%)		9 (13.2%)	3 (4.4%)	
Yes	92 (56.4%)	44 (27%)		37 (54.4%)	19 (27.9%)	
ACE-I/ARB, *n* (%)			0.529			0.973
No	16 (9.8%)	10 (6.1%)		3 (4.4%)	2 (2.9%)	
Yes	93 (57.1%)	44 (27%)		43 (63.2%)	20 (29.4%)	
Beta blocker, *n* (%)			0.809			0.508
No	24 (14.7%)	11 (6.7%)		6 (8.8%)	5 (7.4%)	
Yes	85 (52.1%)	43 (26.4%)		40 (58.8%)	17 (25%)	
MRA, *n* (%)			0.875			0.615
No	37 (22.7%)	19 (11.7%)		10 (14.7%)	6 (8.8%)	
Yes	72 (44.2%)	35 (21.5%)		36 (52.9%)	16 (23.5%)	
Hemoglobin (g/L), mean ± sd	124.1 ± 29.1	122.6 ± 28.3	0.764	132.0 ± 28.0	117.14 ± 25.7	0.039
Creatinine (μmol/L), median (IQR)	94.5 (71.3, 116.7)	85.5 (69.1, 115.6)	0.458	84.1 (71.6, 102.0)	103.5 (78.5, 146.2)	0.049
Urine uric acid (μmol/L), mean ± sd	430.9 ± 148.3	415.3 ± 185.7	0.563	453.2 ± 140.0	414.1 ± 146.1	0.291
LDL (mmol/L), median (IQR)	1.96 (1.42, 2.51)	2.10 (1.39, 2.69)	0.425	2.23 (1.74, 2.86)	1.94 (1.29, 2.65)	0.294
TCH (mmol/L), mean ± sd	3.35 ± 1.17	3.58 ± 1.26	0.267	3.84 ± 1.37	3.66 ± 1.23	0.590
kalium (mmol/L), mean ± sd	3.71 ± 0.50	3.76 ± 0.48	0.529	3.87 ± 0.40	3.82 ± 0.40	0.588
Sodium (mmol/L), median (IQR)	141.6 (139.5, 143.1)	141.8 (139.7, 143.7)	0.703	141.6 (140.3, 143.0)	141.7 (140.3, 142.8)	0.665
NT-proBNP (pg./mL), median (IQR)	2,159 (1,056, 4,556)	2,965 (1,217, 6,891)	0.174	2,610 (655, 5,422)	2,502 (1,083, 5,940)	0.598
FBG, median (IQR)	5.65 (4.76, 6.87)	5.34 (4.77, 6.55)	0.554	5.68 (4.95, 7.96)	5.13 (4.84, 7.52)	0.656
HGS (kg), mean ± sd	35.5 ± 10.5	27.2 ± 9.2	*p* < 0.001	36.8 ± 8.6	27.7 ± 10.1	*p* < 0.001
FFM, mean ± sd	49.9 ± 11.5	42.1 ± 7.6	*p* < 0.001	52.0 ± 9.9	40.1 ± 6.4	*p* < 0.001
FFMI, mean ± sd	17.5 ± 2.8	14.9 ± 1.7	*p* < 0.001	17.7 ± 2.2	14.7 ± 1.8	*p* < 0.001
MUAC (cm), median (IQR)	25.6 (22.3, 30)	19.5 (18.7, 21.9)	*p* < 0.001	26.5 (21.8, 31.0)	20.1 (18.7, 21.4)	*p* < 0.001
PhA, mean ± sd	5.65 ± 0.96	5.36 ± 0.95	0.067	5.59 ± 0.68	5.13 ± 0.99	0.054
Albumin (g/dL), mean ± sd	3.79 ± 0.59	3.54 ± 0.58	0.010	3.81 ± 0.67	3.43 ± 0.71	0.035

ACE-I/ARB, angiotensin converting enzyme inhibitor/angiotensin II receptor blocker; ASMI, appendicular skeletal muscle mass index; BMI, body mass index; NT-proBNP, N-Terminal Pro-Brain Natriuretic Peptide; BUN, blood urea nitrogen; COPD, chronic obstructive pulmonary disease; GLIM, Global Leadership Initiative on Malnutrition; HFmrEF, heart failure with mid-range ejection fraction; HFpEF, heart failure with preserved ejection fraction; HFrEF, heart failure with reduced ejection fraction; LVEF, left ventricular ejection fraction MRA, mineralocorticoid receptor antagonist; NYHA, New York Heart Association. FBG, fasting blood glucose; TCH, total cholesterol.

### 3.2 Independent predictors of malnutrition

**Figure 1:**
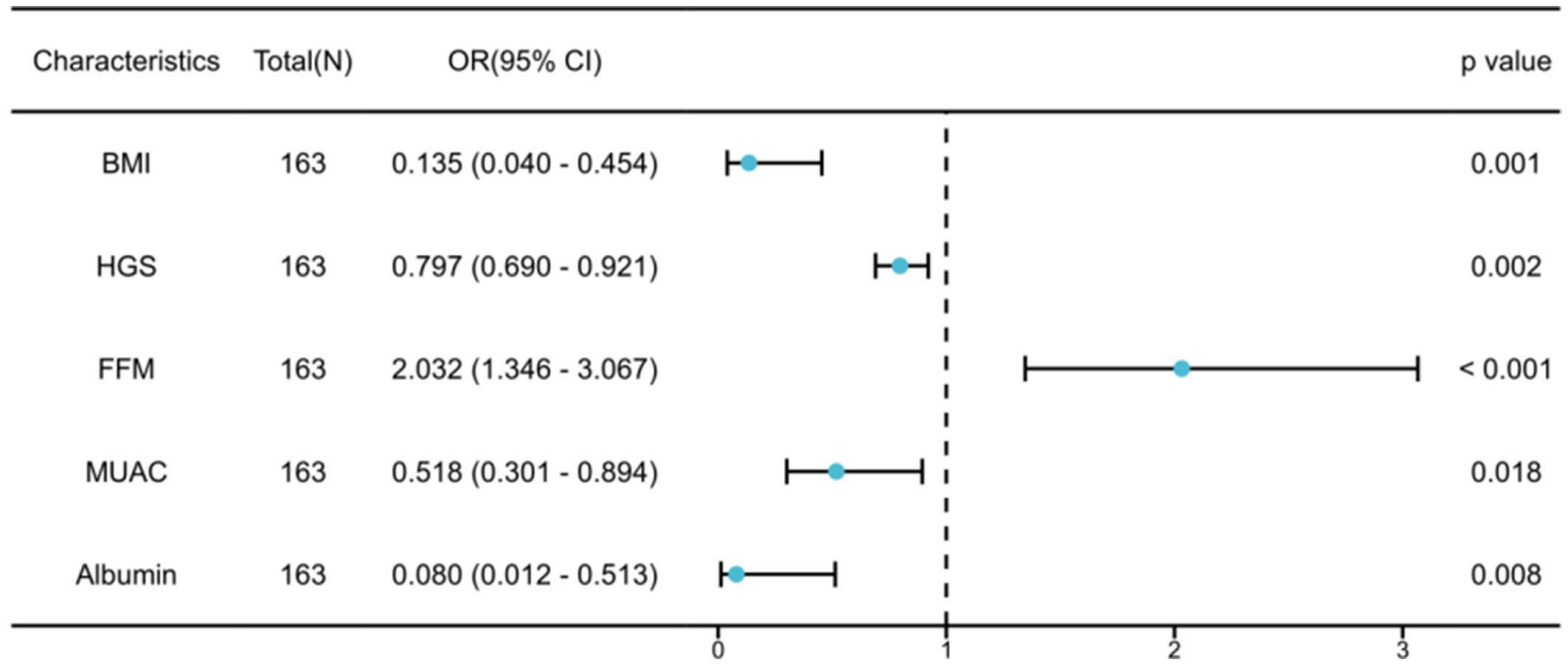
Multivariable logistic regression analysis for malnutrition in older adult heart failure patients.

In Table 2, after conducting univariable logistic regression analysis on the primary cohort, it was found that BMI, ASMI, LVEF, grip strength, NT-proBNP, FFM, fat-free mass index (FFMI), MUAC, whole-body phase angle, and albumin may serve as independent risk factors for malnutrition in older adult HF patients (*p* < 0.05). These variables were further subjected to multivariable logistic regression analysis and, following stepwise variable selection, five variables were identified: BMI (OR: 0.135; 95% CI: 0.040–0.454), HGS (OR: 0.797; 95% CI: 0.690–0.921), FFM (OR: 2.032; 95% CI: 1.346–3.067), MUAC (OR: 0.518; 95% CI: 0.301–0.894), and albumin (OR: 0.080; 95% CI: 0.012–0.513). These factors were identified as independent risk factors of malnutrition in older adult HF patients (*p* < 0.05). To demonstrate the importance and accuracy of the predictive variables, the forest plot was created (as shown in Figure 1).

**Table 2:** Univariable and multivariable logistic regression analyses on factors associated with malnutrition in primary cohort.

Characteristics	Total (*N*)	Univariate analysis		Multivariate analysis	
		OR (95% CI)	*p* value	OR (95% CI)	*p* value
BMI	163	0.537 (0.437–0.660)	< 0.001	0.135 (0.040–0.454)	**0.001**
ASMI	163	0.712 (0.559–0.906)	0.006	1.251 (0.885–1.768)	0.205
LVEF (%)	163	0.963 (0.936–0.990)	0.007	0.851 (0.706–1.027)	0.092
Heart failure phenotypes	163				
HFpEF	83	Reference		Reference	
HFmrEF	35	1.861 (0.795–4.357)	0.152	0.240 (0.009–6.472)	0.396
HFrEF	45	2.756 (1.274–5.965)	0.010	0.027 (0.000–5.805)	0.188
NT-proBNP	163	1.000 (1.000–1.000)	0.033	1.000 (1.000–1.000)	0.693
HGS	163	0.925 (0.893–0.958)	< 0.001	0.797 (0.690–0.921)	**0.002**
FFM	163	0.929 (0.896–0.962)	< 0.001	2.032 (1.346–3.067)	**< 0.001**
FFMI	163	0.629 (0.527–0.750)	< 0.001	1.036 (0.240–4.482)	0.962
MUAC	163	0.738 (0.659–0.826)	< 0.001	0.518 (0.301–0.894)	**0.018**
PhA	163	0.721 (0.506–1.027)	0.070	0.857 (0.265–2.767)	0.796
Albumin	163	0.473 (0.264–0.846)	0.012	0.080 (0.012–0.513)	**0.008**

ASMI, appendicular skeletal muscle mass index; BMI, body mass index; NT-proBNP, N-Terminal Pro-Brain Natriuretic Peptide; HFmrEF, heart failure with mid-range ejection fraction; HFpEF, heart failure with preserved ejection fraction; HFrEF, heart failure with reduced ejection fraction; LVEF, left ventricular ejection fraction. *p*-values less than 0.05 are emphasized in boldface.

### 3.3 Development and assessment of the nomogram

**Figure 2:**
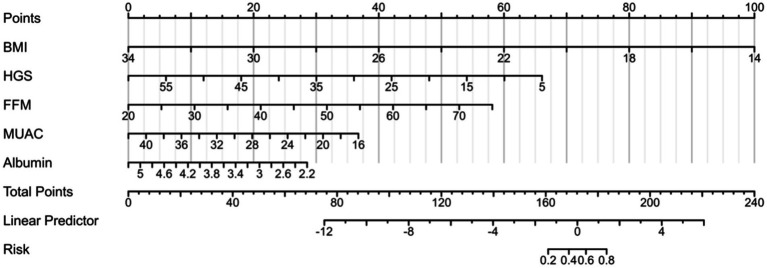
Establishment of the nomogram for predicting malnutrition in older adult heart failure patients. The nomogram-predicted probability of malnutrition is plotted on the x-axis the actual risk of residual lesions is plotted on the y-axis.

**Figure 3:**
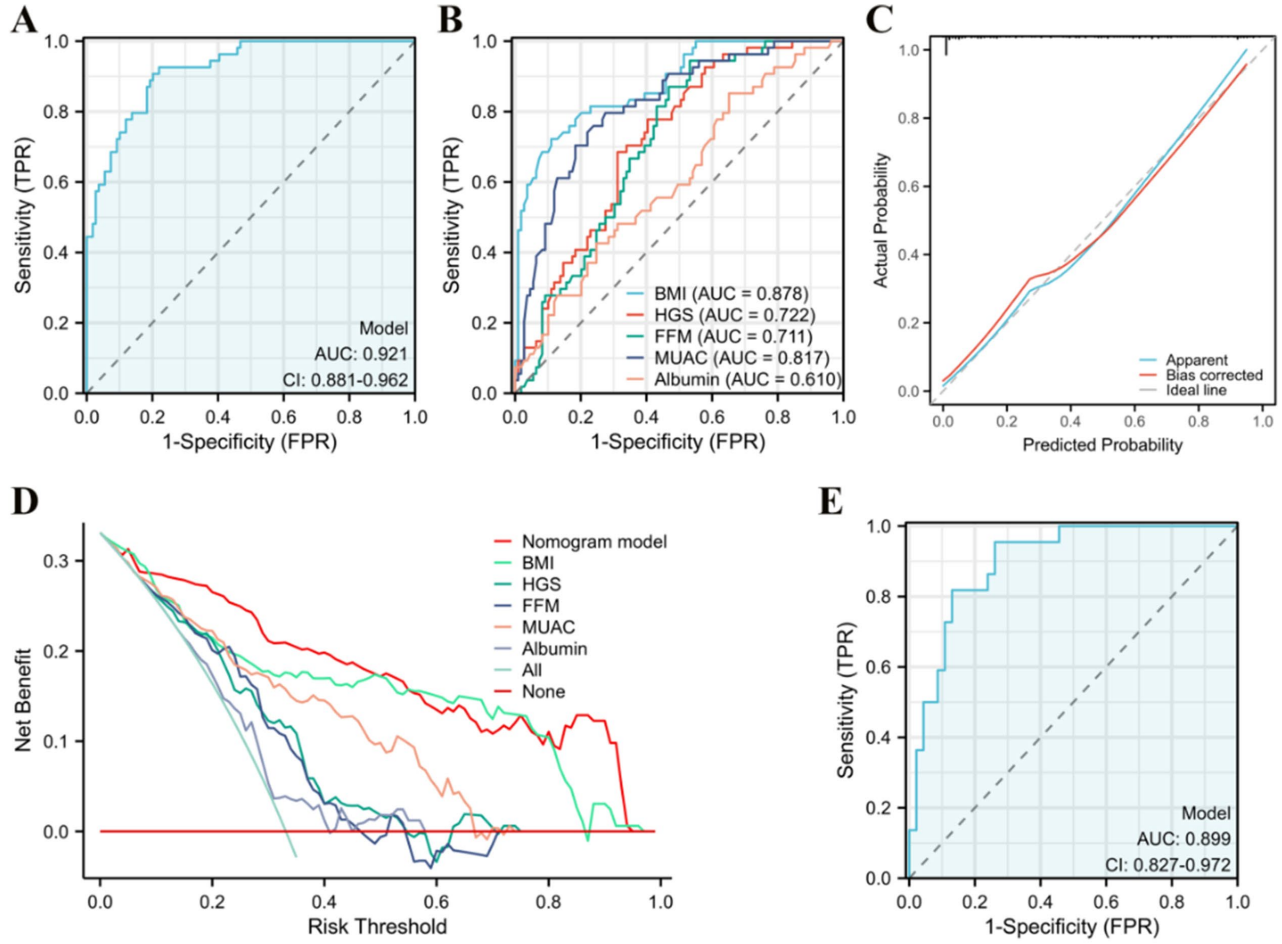
Evaluation of the nomogram. **(A)** Area under the ROC curves (AUC) for the diagnosis of malnutrition using the nomogram in older adult heart failure patients. **(B)** AUC for the diagnosis of malnutrition using single indicator. **(C)** The calibration curve for the risk of malnutrition. **(D)** Decision curve analysis of the nomogram (red line), **(E)** AUC for the diagnosis of malnutrition by the nomogram in the validation queue.

The five predetermined independent predictive factors were incorporated into a nomogram aimed at predicting malnutrition (Figure 2). The training dataset underwent 5-fold cross-validation repeated 1,000 times for internal validation, resulting in a C-index of 0.921. Subsequently, the model’s diagnostic performance was evaluated by plotting the Receiver Operating Characteristic (ROC) curve and calculating the AUC, alongside drawing a calibration curve to assess the nomogram model’s consistency. The nomogram demonstrated an AUC value of 0.921 (95% CI: 0.881–0.962) as shown in Figure 3A, significantly surpassing the diagnostic accuracy of using any single indicator for identifying malnutrition in older adult HF patients (Figure 3B). The calibration curve of the nomogram, depicting malnutrition probability, displayed a strong agreement between predicted and observed outcomes (Figure 3C). Validation of the nomogram for diagnosing the cohort and plotting the ROC curve (Figure 3E) revealed the AUC of 0.889, 95% CI: 0.827–0.972, highlighting the significant clinical usefulness of the nomogram model. In conclusion, the nomogram demonstrates a robust ability to discriminate and calibrate effectively in diagnosing malnutrition in older adult heart failure patients.

### 3.4 Clinical benefit of the nomogram

Decision curve analysis (DCA) was used to evaluate the clinical utility of the model, and the results are depicted in Figure 3D. As per the decision curve, if a specific patient’s threshold probability is >0, utilizing the nomogram for predicting malnutrition yields more benefits than treating all patients or adopting no treatment plan. Furthermore, when compared to single indicators such as BMI, HGS, FFM, MUAC, and albumin for diagnosis, the nomogram offers greater utility across all threshold probability values. The findings suggest that, with GLIM as the diagnostic gold standard, the nomogram model demonstrates noteworthy clinical utility in diagnosing malnutrition among older adult HF patients.

## 4 Discussion

The analysis of disease risk factors and the establishment of corresponding prediction models provide new insights for disease prevention and management (18–20). In this study, the GLIM criteria were employed to diagnose malnutrition in older adult HF patients. The findings revealed that BMI, HGS, FFM, MUAC, and serum albumin levels are significant independent risk factors for malnutrition in this population. We established a nomogram prediction model using these 5 independent risk factors as predictive indicators for diagnosing malnutrition in older adult HF patients, and confirmed that this is a simple, rapid, and accurate method for diagnosing malnutrition specifically in older adult HF patients.

### 4.1 The incidence rate of malnutrition

Currently, there is no standardized method for diagnosing malnutrition in older adult heart failure patients (3). Using diverse risk screening tools instead of relying on consensus diagnostic criteria can potentially result in misdiagnosis and complicate the assessment of whether patients would benefit from nutritional intervention (21). The GLIM criteria amalgamate evidence and expert opinions from the prominent enteral and parenteral nutrition societies worldwide, including those from Europe, America, Asia, and Latin America (8). The effectiveness of diagnosing malnutrition is gradually being validated in various diseases, with the potential to become the global standard for malnutrition diagnosis. In this study, according to the GLIM criteria, the prevalence of malnutrition among older adult HF patients was 33.1% in the primary cohort and 32.4% in the validation cohort, consistent with prior research findings (12, 22).

### 4.2 The advantages of nomogram model

In accordance with current expert consensus, although the GLIM standard has implemented a two-step approach (8), the process and assessment of corresponding indicators remain relatively complex. Accordingly, we conducted an analysis based on the GLIM diagnosis and devised a simpler and more efficient nomogram model. This model enables rapid and straightforward prediction of malnutrition and offers a quantitative display. Using GLIM as the benchmark, it yielded an AUC value of 0.921, with a 95% CI of 0.881–0.962, for distinguishing malnourished older adult HF patients. In the validation cohort test, the AUC value was 0.889, with a 95%CI ranging from 0.827 to 0.972. The nomogram model demonstrates high performance in predicting malnutrition in older adult HF patients. Furthermore, previous studies have demonstrated that, compared to the GLIM standard, the accuracy of diagnosing malnutrition in heart failure patients using the Mini Nutritional Assessment (MNA) method is a mere 77.4% (23). In contrast, the accuracy using the MUST tool is only 80% (24), while the diagnostic accuracy of the ESPEN standard is significantly lower at 46.6% (11). These findings suggest that our nomogram model may offer superior diagnostic efficacy compared to the aforementioned measures.

Five indicators are involved in the nomogram model: BMI, HGS, FFM, MUAC, and albumin. BMI serves as a crucial measure for assessing malnutrition, with the World Health Organization defining a BMI < 18.5 kg/m2 as the diagnostic threshold for malnutrition (25). Nevertheless, this criterion overlooks the changes in body composition resulting from malnutrition. In 2015, ESPEN introduced BMI, unintentional weight loss, and FFMI as crucial diagnostic parameters for identifying malnutrition, thereby improving diagnostic accuracy (26). FFMI serves as a critical indicator of body composition (27), frequently employed in the clinical diagnosis of sarcopenia. Its inclusion in the diagnosis of malnutrition underscores the essential role of body composition in evaluating malnutrition. After analysis, we included the FFM as an indicator in the model, as the multivariable logistic regression revealed an OR of 2.032 (95% CI: 1.346–3.067, *p* < 0.001). FMM is a body composition estimate that is frequently reported in both research studies and clinical settings. However, to date, no research has utilized it in malnutrition assessment models specifically designed for patients with heart failure (28). Recent research suggests that the use of the GLIM standard for diagnosing malnutrition provides superior guidance for clinical practice compared to the ESPEN standard (29, 30). A recent study revealed that the GLIM criteria exhibited a higher accuracy in diagnosing malnutrition in patients with Crohn’s disease compared to the ESPEN criteria (84% vs. 79%) (13). Additionally, among older adult hospitalized patients, the malnutrition detection rate using the ESPEN criteria was less than half that of the GLIM criteria (17% vs. 37.8%) (31). Studies in cardiovascular patients have confirmed that malnutrition defined according to the GLIM standard better reflects the nutritional status of CVD patients and helps predict the prognosis of CVD patients (11). Hence, we employ the GLIM standard as the diagnostic criterion and perform logistic regression analysis. Handgrip strength (HGS) and albumin levels have been consistently linked to malnutrition in HF patients (32–35). HGS is a reliable indicator of muscle function in patients with heart failure and serves as an important measure for assessing their nutritional status (36). Albumin, synthesized by hepatocytes and regulated by various stimuli—primarily nutritional intake—is also a significant marker of nutritional status in these patients; however, its levels can be easily affected by measurement factors (37). Furthermore, mid-upper arm circumference (MUAC) has been associated with malnutrition and adverse clinical outcomes in older adult patients (16). MUAC can also reflect the lean muscle condition of older adult heart failure patients, demonstrating a strong complementary relationship with BMI (38, 39).

The above indicators can be obtained through routine admission blood tests and simple anthropometric measurements, offering cost-effective solutions. This straightforward and efficient diagnostic model provides significant convenience for dietitians and clinical physicians, particularly during outpatient visits, enhancing the efficiency of evaluating malnutrition in older adult HF patients. It facilitates guidance for subsequent nutritional therapies. Additionally, in regions with limited laboratory technology or in remote areas or countries, the nomogram model will play a crucial role.

### 4.3 Limitations

This study has several limitations. Firstly, while the nomogram model effectively diagnoses malnutrition, it lacks the capability to differentiate the severity of malnutrition. Secondly, considering prior research on the influence of gender differences on the nutritional status and prognosis of heart failure patients (40, 41), we lack a discussion on the relationship between gender differences and the nutritional status and diagnostic models of heart failure patients. In the forthcoming follow-up process, we will closely monitor the effects of gender differences on the nutritional status and prognosis of heart failure patients. Thirdly, the model has not yet been developed into web-based or application software (42), which limits its convenience for clinical use; future improvements will be made in this regard. Finally, since the data for this study originate from a single center, further multi-center studies are necessary to validate the accuracy and efficacy of the model.

## 5 Conclusion

Based on data from training and validation queues, this study reveals that BMI, HGS, FFM, MUAC, and albumin are independent risk factors associated with malnutrition in older adult HF patients. A rapid and efficient diagnostic nomogram model for malnutrition was developed and validated to facilitate the convenient diagnosis of malnutrition in older adult HF patients, providing valuable insights for clinical decision-making.

## Data Availability

The original contributions presented in the study are included in the article/supplementary material, further inquiries can be directed to the corresponding author.
